# Ethyl 2-(4-bromophenyl)-1-[3-(1*H*-imidazol-1-yl)prop­yl]-1*H*-benzimidazole-5-carboxyl­ate monohydrate

**DOI:** 10.1107/S160053681104342X

**Published:** 2011-10-29

**Authors:** Yeong Keng Yoon, Mohamed Ashraf Ali, Tan Soo Choon, Madhukar Hemamalini, Hoong-Kun Fun

**Affiliations:** a Institute for Research in Molecular Medicine, Universiti Sains Malaysia, 11800 USM, Penang, Malaysia; b X-ray Crystallography Unit, School of Physics, Universiti Sains Malaysia, 11800 USM, Penang, Malaysia

## Abstract

In the title compound, C_22_H_21_BrN_4_O_2_·H_2_O, the two pyrazole rings are essentially planar [maximum deviations 0.002 (1) and 0.002 (1) Å], and form a dihedral angle of 73.46 (9)°. The dihedral angle between the benzene rings is 29.33 (7)°. In the crystal, mol­ecules are connected *via* C—H⋯O and O—H⋯N hydrogen bonds, forming layers in the *ab* plane.

## Related literature

For applications of benzimidazole derivatives, see: Garuti *et al.* (2000 ▶); Rao *et al.* (2002 ▶); Thakurdesai *et al.* (2007 ▶); Yoon *et al.* (2011 ▶). For the stability of the temperature controller used in the data collection, see: Cosier & Glazer (1986 ▶).
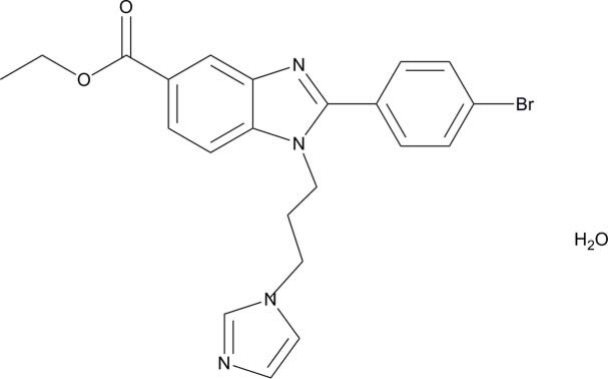


## Experimental

### 

#### Crystal data

C_22_H_21_BrN_4_O_2_·H_2_O
*M* *_r_* = 471.35
Monoclinic, 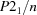

*a* = 9.1854 (1) Å
*b* = 16.7389 (2) Å
*c* = 13.7379 (2) Å
β = 98.283 (1)°
*V* = 2090.22 (5) Å^3^
*Z* = 4
Mo *K*α radiation
μ = 2.00 mm^−1^
*T* = 100 K
0.47 × 0.42 × 0.41 mm

#### Data collection

Bruker SMART APEXII CCD area-detector diffractometer
Absorption correction: multi-scan (*SADABS*; Bruker, 2009 ▶) *T*_min_ = 0.452, *T*_max_ = 0.494
28847 measured reflections
7482 independent reflections
5514 reflections with *I* > 2σ(*I*)
*R*_int_ = 0.032

#### Refinement

*R*[*F* ^2^ > 2σ(*F* ^2^)] = 0.037
*wR*(*F* ^2^) = 0.085
*S* = 1.03
7482 reflections
280 parameters
H atoms treated by a mixture of independent and constrained refinement
Δρ_max_ = 0.63 e Å^−3^
Δρ_min_ = −0.34 e Å^−3^

### 

Data collection: *APEX2* (Bruker, 2009 ▶); cell refinement: *SAINT* (Bruker, 2009 ▶); data reduction: *SAINT*; program(s) used to solve structure: *SHELXTL* (Sheldrick, 2008 ▶); program(s) used to refine structure: *SHELXTL*; molecular graphics: *SHELXTL*; software used to prepare material for publication: *SHELXTL* and *PLATON* (Spek, 2009 ▶).

## Supplementary Material

Crystal structure: contains datablock(s) global, I. DOI: 10.1107/S160053681104342X/tk5002sup1.cif

Structure factors: contains datablock(s) I. DOI: 10.1107/S160053681104342X/tk5002Isup2.hkl

Supplementary material file. DOI: 10.1107/S160053681104342X/tk5002Isup3.cml

Additional supplementary materials:  crystallographic information; 3D view; checkCIF report

## Figures and Tables

**Table 1:** Hydrogen-bond geometry (Å, °)

*D*—H⋯*A*	*D*—H	H⋯*A*	*D*⋯*A*	*D*—H⋯*A*
O1*W*—H2*W*1⋯N4^i^	0.92 (3)	1.99 (3)	2.910 (2)	175 (3)
O1*W*—H1*W*1⋯N1^ii^	0.81 (3)	2.16 (3)	2.891 (2)	151 (2)
C17—H17*B*⋯O1*W*^iii^	0.99	2.41	3.236 (2)	141
C19—H19*B*⋯O1*W*^iii^	0.99	2.56	3.327 (2)	135
C20—H20*A*⋯O2^iv^	0.95	2.58	3.301 (2)	133

Symmetry codes: (i) 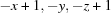; (ii) 
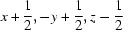
; (iii) 
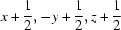
; (iv) 
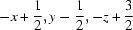
.

**Fig. 1.**
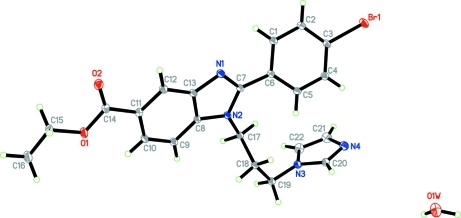
The asymmetric unit of the title compound, showing 30% probability displacement ellipsoids.

## supplementary crystallographic information

### Comment

Benzimidazole derivatives are of wide interest because of their diverse biological activities and various clinical applications. Benzimidazoles are a class of bioactive heterocyclic compounds which exhibit a wide range of activities such as anti-proliferative (Garuti *et al.*, 2000), anti-HIV (Rao *et al.*, 2002), anti-inflammatory and anthelmintic (Thakurdesai *et al.*, 2007) properties. As part of our on-going structural studies of benzimidazole derivatives (Yoon *et al.*, 2011), we now report the structure of the title compound.

In the title compound (Fig. 1), the two pyrazole (N1,N2/C7,C8/C13 and N3,N4/C20–C22) rings are essentially planar, with a maximum deviation of 0.002 (1) Å for atom C8 and 0.002 (1) Å for atom N3. The dihedral angle between the two pyrazole (N1,N2/C7,C8/C13 : N3,N4/C20–C22) rings is 73.46 (9)° and between the two benzene (C8–C13 : C1–C6) rings is 29.33 (7)°.

In the crystal structure, molecules are connected *via* intermolecular C—H···O and O—H···N (Table 1) hydrogen bonds, forming layers in the *ab* plane.

### Experimental

Ethyl-4-(3-(1*H*-imidazol-1-yl-propylamino)-3-aminobenzoate (0.84 mmol) and sodium metabisulfite adduct of bromobenzaldehyde (1.68 mmol) were dissolved in DMF. The reaction mixture was refluxed at 130°C for 2 h. After completion, the reaction mixture was diluted in ethyl acetate (20 ml) and washed with water (20 ml). The organic layer was collected, dried over Na_2_SO_4_ and then evaporated *in vacuo* to yield the product. The product was recrystallised from its ethyl acetate solution.

### Refinement

Atoms H2W1 and H1W1 were located from a difference Fourier maps and refined freely [O—H = 0.80 (3)–0.92 (3) Å]. The remaining H atoms were positioned geometrically [C—H = 0.95–0.99 Å] and were refined using a riding model, with *U*_iso_(H) = 1.2 or 1.5*U*_eq_(C). A rotating group model was applied to the methyl group.

### Crystal data

**Table:**

C_22_H_21_BrN_4_O_2_·H_2_O	*F*(000) = 968
*M**_r_* = 471.35	*D*_x_ = 1.498 Mg m^−^^3^
Monoclinic, *P*2_1_/*n*	Mo *K*α radiation, λ = 0.71073 Å
Hall symbol: -P 2yn	Cell parameters from 9939 reflections
*a* = 9.1854 (1) Å	θ = 2.5–31.3°
*b* = 16.7389 (2) Å	µ = 2.00 mm^−^^1^
*c* = 13.7379 (2) Å	*T* = 100 K
β = 98.283 (1)°	Block, yellow
*V* = 2090.22 (5) Å^3^	0.47 × 0.42 × 0.41 mm
*Z* = 4	

### Data collection

**Table:**

Bruker SMART APEXII CCD area-detector diffractometer	7482 independent reflections
Radiation source: fine-focus sealed tube	5514 reflections with *I* > 2σ(*I*)
graphite	*R*_int_ = 0.032
φ and ω scans	θ_max_ = 32.4°, θ_min_ = 1.9°
Absorption correction: multi-scan (*SADABS*; Bruker, 2009)	*h* = −13→13
*T*_min_ = 0.452, *T*_max_ = 0.494	*k* = −25→17
28847 measured reflections	*l* = −20→19

### Refinement

**Table:**

Refinement on *F*^2^	Primary atom site location: structure-invariant direct methods
Least-squares matrix: full	Secondary atom site location: difference Fourier map
*R*[*F*^2^ > 2σ(*F*^2^)] = 0.037	Hydrogen site location: inferred from neighbouring sites
*wR*(*F*^2^) = 0.085	H atoms treated by a mixture of independent and constrained refinement
*S* = 1.03	*w* = 1/[σ^2^(*F*_o_^2^) + (0.0386*P*)^2^ + 0.518*P*] where *P* = (*F*_o_^2^ + 2*F*_c_^2^)/3
7482 reflections	(Δ/σ)_max_ = 0.001
280 parameters	Δρ_max_ = 0.63 e Å^−^^3^
0 restraints	Δρ_min_ = −0.34 e Å^−^^3^

### Special details

**Table:**

Experimental. The crystal was placed in the cold stream of an Oxford Cryosystems Cobra open-flow nitrogen cryostat (Cosier & Glazer, 1986) operating at 100.0 (1) K.
Geometry. All s.u.'s (except the s.u. in the dihedral angle between two l.s. planes) are estimated using the full covariance matrix. The cell s.u.'s are taken into account individually in the estimation of s.u.'s in distances, angles and torsion angles; correlations between s.u.'s in cell parameters are only used when they are defined by crystal symmetry. An approximate (isotropic) treatment of cell s.u.'s is used for estimating s.u.'s involving l.s. planes.
Refinement. Refinement of F^2^ against ALL reflections. The weighted R-factor wR and goodness of fit S are based on F^2^, conventional R-factors R are based on F, with F set to zero for negative F^2^. The threshold expression of F^2^ > 2σ(F^2^) is used only for calculating R-factors(gt) etc. and is not relevant to the choice of reflections for refinement. R-factors based on F^2^ are statistically about twice as large as those based on F, and R- factors based on ALL data will be even larger.

### Fractional atomic coordinates and isotropic or equivalent isotropic displacement parameters (Å^2^)

**Table:**

	*x*	*y*	*z*	*U*_iso_*/*U*_eq_	
Br1	0.123446 (18)	0.050021 (9)	0.931417 (13)	0.02720 (6)	
O1	0.40323 (12)	0.80153 (6)	0.87745 (9)	0.0247 (2)	
O2	0.17930 (13)	0.76409 (7)	0.90802 (9)	0.0260 (2)	
N1	0.23105 (14)	0.45313 (7)	0.89922 (10)	0.0199 (3)	
N2	0.46106 (14)	0.42561 (7)	0.87069 (9)	0.0174 (2)	
N3	0.71289 (14)	0.23427 (8)	0.76584 (10)	0.0216 (3)	
N4	0.61875 (16)	0.11193 (8)	0.75103 (11)	0.0265 (3)	
C1	0.18138 (17)	0.29413 (9)	0.95735 (11)	0.0207 (3)	
H1A	0.1411	0.3363	0.9913	0.025*	
C2	0.13384 (17)	0.21676 (9)	0.96832 (12)	0.0209 (3)	
H2A	0.0622	0.2056	1.0099	0.025*	
C3	0.19242 (17)	0.15558 (9)	0.91768 (11)	0.0197 (3)	
C4	0.29838 (17)	0.17037 (9)	0.85769 (12)	0.0212 (3)	
H4A	0.3385	0.1279	0.8242	0.025*	
C5	0.34509 (17)	0.24861 (9)	0.84737 (11)	0.0201 (3)	
H5A	0.4170	0.2595	0.8059	0.024*	
C6	0.28788 (16)	0.31134 (8)	0.89703 (11)	0.0174 (3)	
C7	0.32652 (16)	0.39632 (9)	0.88789 (11)	0.0181 (3)	
C8	0.44933 (16)	0.50829 (8)	0.87200 (11)	0.0175 (3)	
C9	0.54989 (17)	0.56882 (9)	0.86054 (12)	0.0207 (3)	
H9A	0.6473	0.5574	0.8490	0.025*	
C10	0.50014 (17)	0.64649 (9)	0.86687 (11)	0.0204 (3)	
H10A	0.5648	0.6895	0.8589	0.025*	
C11	0.35573 (17)	0.66335 (8)	0.88480 (11)	0.0188 (3)	
C12	0.25683 (17)	0.60237 (9)	0.89609 (11)	0.0201 (3)	
H12A	0.1595	0.6137	0.9078	0.024*	
C13	0.30541 (16)	0.52409 (9)	0.88960 (11)	0.0186 (3)	
C14	0.30222 (17)	0.74678 (9)	0.89169 (11)	0.0209 (3)	
C15	0.35706 (19)	0.88442 (9)	0.88439 (14)	0.0271 (4)	
H15A	0.3201	0.8936	0.9477	0.033*	
H15B	0.2771	0.8970	0.8302	0.033*	
C16	0.4876 (2)	0.93640 (10)	0.87762 (16)	0.0345 (4)	
H16A	0.4609	0.9924	0.8859	0.052*	
H16B	0.5196	0.9293	0.8131	0.052*	
H16C	0.5679	0.9215	0.9294	0.052*	
C17	0.59623 (16)	0.38267 (9)	0.85909 (11)	0.0186 (3)	
H17A	0.5934	0.3290	0.8891	0.022*	
H17B	0.6814	0.4118	0.8948	0.022*	
C18	0.61756 (17)	0.37351 (9)	0.75149 (12)	0.0209 (3)	
H18A	0.5262	0.3523	0.7132	0.025*	
H18B	0.6376	0.4265	0.7242	0.025*	
C19	0.74492 (18)	0.31714 (9)	0.74141 (13)	0.0241 (3)	
H19A	0.7669	0.3193	0.6730	0.029*	
H19B	0.8335	0.3354	0.7854	0.029*	
C20	0.62362 (17)	0.18286 (9)	0.70936 (12)	0.0223 (3)	
H20A	0.5706	0.1962	0.6469	0.027*	
C21	0.7101 (2)	0.11857 (11)	0.83933 (14)	0.0318 (4)	
H21A	0.7297	0.0769	0.8864	0.038*	
C22	0.76797 (19)	0.19311 (11)	0.84941 (13)	0.0310 (4)	
H22A	0.8338	0.2130	0.9037	0.037*	
O1W	0.41413 (16)	0.03202 (9)	0.36883 (13)	0.0409 (4)	
H2W1	0.406 (4)	−0.015 (2)	0.334 (2)	0.092 (10)*	
H1W1	0.496 (3)	0.0391 (14)	0.3976 (19)	0.052 (8)*	

### Atomic displacement parameters (Å^2^)

**Table:**

	*U*^11^	*U*^22^	*U*^33^	*U*^12^	*U*^13^	*U*^23^
Br1	0.02447 (9)	0.01647 (7)	0.04180 (11)	−0.00321 (6)	0.00871 (7)	0.00484 (6)
O1	0.0236 (6)	0.0141 (5)	0.0369 (7)	−0.0002 (4)	0.0064 (5)	0.0000 (4)
O2	0.0224 (6)	0.0207 (5)	0.0355 (7)	0.0011 (4)	0.0059 (5)	−0.0032 (5)
N1	0.0168 (6)	0.0168 (6)	0.0267 (7)	−0.0011 (5)	0.0054 (5)	0.0010 (5)
N2	0.0152 (6)	0.0147 (5)	0.0232 (6)	−0.0008 (4)	0.0056 (5)	0.0004 (5)
N3	0.0179 (6)	0.0207 (6)	0.0270 (7)	0.0007 (5)	0.0065 (5)	−0.0022 (5)
N4	0.0254 (7)	0.0227 (7)	0.0324 (8)	0.0012 (5)	0.0077 (6)	0.0011 (6)
C1	0.0190 (7)	0.0200 (7)	0.0239 (8)	−0.0014 (6)	0.0056 (6)	−0.0009 (6)
C2	0.0179 (7)	0.0215 (7)	0.0239 (8)	−0.0016 (6)	0.0054 (6)	0.0029 (6)
C3	0.0187 (7)	0.0157 (6)	0.0245 (8)	−0.0022 (5)	0.0025 (6)	0.0041 (6)
C4	0.0213 (8)	0.0178 (7)	0.0250 (8)	0.0001 (6)	0.0051 (6)	0.0002 (6)
C5	0.0190 (7)	0.0193 (7)	0.0232 (8)	−0.0012 (5)	0.0070 (6)	0.0010 (6)
C6	0.0158 (7)	0.0170 (6)	0.0197 (7)	−0.0021 (5)	0.0032 (5)	0.0013 (5)
C7	0.0178 (7)	0.0172 (6)	0.0198 (7)	−0.0014 (5)	0.0039 (5)	0.0003 (5)
C8	0.0180 (7)	0.0149 (6)	0.0199 (7)	−0.0001 (5)	0.0039 (5)	−0.0004 (5)
C9	0.0176 (7)	0.0197 (7)	0.0255 (8)	−0.0019 (5)	0.0056 (6)	0.0000 (6)
C10	0.0205 (7)	0.0174 (6)	0.0237 (8)	−0.0035 (6)	0.0042 (6)	0.0011 (6)
C11	0.0199 (7)	0.0164 (6)	0.0199 (7)	−0.0001 (5)	0.0023 (6)	−0.0006 (5)
C12	0.0169 (7)	0.0196 (7)	0.0238 (8)	0.0003 (5)	0.0031 (6)	0.0003 (6)
C13	0.0166 (7)	0.0172 (6)	0.0221 (7)	−0.0019 (5)	0.0035 (6)	0.0008 (5)
C14	0.0214 (8)	0.0186 (7)	0.0221 (8)	−0.0010 (6)	0.0007 (6)	−0.0014 (6)
C15	0.0292 (9)	0.0142 (7)	0.0384 (10)	0.0025 (6)	0.0062 (7)	−0.0004 (6)
C16	0.0313 (10)	0.0184 (8)	0.0557 (12)	−0.0001 (6)	0.0127 (9)	−0.0008 (7)
C17	0.0162 (7)	0.0175 (6)	0.0229 (7)	0.0004 (5)	0.0055 (6)	0.0002 (5)
C18	0.0208 (7)	0.0183 (7)	0.0250 (8)	−0.0012 (6)	0.0079 (6)	−0.0007 (6)
C19	0.0208 (8)	0.0213 (7)	0.0325 (9)	−0.0039 (6)	0.0113 (6)	−0.0039 (6)
C20	0.0211 (8)	0.0215 (7)	0.0250 (8)	−0.0002 (6)	0.0063 (6)	−0.0018 (6)
C21	0.0301 (9)	0.0293 (9)	0.0351 (10)	0.0053 (7)	0.0013 (8)	0.0065 (7)
C22	0.0259 (9)	0.0338 (9)	0.0313 (9)	0.0032 (7)	−0.0027 (7)	0.0001 (7)
O1W	0.0218 (7)	0.0354 (8)	0.0648 (10)	0.0047 (6)	0.0035 (7)	−0.0090 (7)

### Geometric parameters (Å, °)

**Table:**

Br1—C3	1.8957 (14)	C9—H9A	0.9500
O1—C14	1.3384 (19)	C10—C11	1.412 (2)
O1—C15	1.4579 (18)	C10—H10A	0.9500
O2—C14	1.2174 (19)	C11—C12	1.390 (2)
N1—C7	1.3179 (19)	C11—C14	1.488 (2)
N1—C13	1.3860 (18)	C12—C13	1.391 (2)
N2—C7	1.3811 (19)	C12—H12A	0.9500
N2—C8	1.3884 (18)	C15—C16	1.495 (2)
N2—C17	1.4630 (19)	C15—H15A	0.9900
N3—C20	1.353 (2)	C15—H15B	0.9900
N3—C22	1.372 (2)	C16—H16A	0.9800
N3—C19	1.4668 (19)	C16—H16B	0.9800
N4—C20	1.322 (2)	C16—H16C	0.9800
N4—C21	1.376 (2)	C17—C18	1.527 (2)
C1—C2	1.382 (2)	C17—H17A	0.9900
C1—C6	1.400 (2)	C17—H17B	0.9900
C1—H1A	0.9500	C18—C19	1.525 (2)
C2—C3	1.389 (2)	C18—H18A	0.9900
C2—H2A	0.9500	C18—H18B	0.9900
C3—C4	1.385 (2)	C19—H19A	0.9900
C4—C5	1.392 (2)	C19—H19B	0.9900
C4—H4A	0.9500	C20—H20A	0.9500
C5—C6	1.396 (2)	C21—C22	1.355 (3)
C5—H5A	0.9500	C21—H21A	0.9500
C6—C7	1.476 (2)	C22—H22A	0.9500
C8—C9	1.395 (2)	O1W—H2W1	0.92 (3)
C8—C13	1.403 (2)	O1W—H1W1	0.80 (3)
C9—C10	1.385 (2)		
			
C14—O1—C15	115.33 (12)	N1—C13—C8	110.14 (13)
C7—N1—C13	105.17 (13)	C12—C13—C8	120.50 (14)
C7—N2—C8	106.18 (12)	O2—C14—O1	123.01 (14)
C7—N2—C17	129.68 (12)	O2—C14—C11	123.97 (14)
C8—N2—C17	124.04 (12)	O1—C14—C11	113.02 (13)
C20—N3—C22	106.29 (14)	O1—C15—C16	107.83 (14)
C20—N3—C19	126.52 (14)	O1—C15—H15A	110.1
C22—N3—C19	127.19 (14)	C16—C15—H15A	110.1
C20—N4—C21	104.76 (14)	O1—C15—H15B	110.1
C2—C1—C6	121.07 (15)	C16—C15—H15B	110.1
C2—C1—H1A	119.5	H15A—C15—H15B	108.5
C6—C1—H1A	119.5	C15—C16—H16A	109.5
C1—C2—C3	119.01 (15)	C15—C16—H16B	109.5
C1—C2—H2A	120.5	H16A—C16—H16B	109.5
C3—C2—H2A	120.5	C15—C16—H16C	109.5
C4—C3—C2	121.48 (14)	H16A—C16—H16C	109.5
C4—C3—Br1	119.95 (12)	H16B—C16—H16C	109.5
C2—C3—Br1	118.56 (12)	N2—C17—C18	112.59 (12)
C3—C4—C5	118.83 (14)	N2—C17—H17A	109.1
C3—C4—H4A	120.6	C18—C17—H17A	109.1
C5—C4—H4A	120.6	N2—C17—H17B	109.1
C4—C5—C6	120.99 (14)	C18—C17—H17B	109.1
C4—C5—H5A	119.5	H17A—C17—H17B	107.8
C6—C5—H5A	119.5	C19—C18—C17	110.93 (13)
C5—C6—C1	118.62 (13)	C19—C18—H18A	109.5
C5—C6—C7	124.85 (14)	C17—C18—H18A	109.5
C1—C6—C7	116.47 (13)	C19—C18—H18B	109.5
N1—C7—N2	113.02 (13)	C17—C18—H18B	109.5
N1—C7—C6	120.87 (13)	H18A—C18—H18B	108.0
N2—C7—C6	126.07 (13)	N3—C19—C18	112.50 (13)
N2—C8—C9	131.96 (14)	N3—C19—H19A	109.1
N2—C8—C13	105.48 (12)	C18—C19—H19A	109.1
C9—C8—C13	122.55 (13)	N3—C19—H19B	109.1
C10—C9—C8	116.42 (14)	C18—C19—H19B	109.1
C10—C9—H9A	121.8	H19A—C19—H19B	107.8
C8—C9—H9A	121.8	N4—C20—N3	112.23 (15)
C9—C10—C11	121.69 (14)	N4—C20—H20A	123.9
C9—C10—H10A	119.2	N3—C20—H20A	123.9
C11—C10—H10A	119.2	C22—C21—N4	110.16 (15)
C12—C11—C10	121.21 (14)	C22—C21—H21A	124.9
C12—C11—C14	117.06 (14)	N4—C21—H21A	124.9
C10—C11—C14	121.73 (13)	C21—C22—N3	106.55 (15)
C11—C12—C13	117.63 (14)	C21—C22—H22A	126.7
C11—C12—H12A	121.2	N3—C22—H22A	126.7
C13—C12—H12A	121.2	H2W1—O1W—H1W1	113 (3)
N1—C13—C12	129.35 (14)		
			
C6—C1—C2—C3	−0.6 (2)	C14—C11—C12—C13	179.87 (13)
C1—C2—C3—C4	0.8 (2)	C7—N1—C13—C12	−179.26 (15)
C1—C2—C3—Br1	−178.80 (11)	C7—N1—C13—C8	0.03 (17)
C2—C3—C4—C5	−0.9 (2)	C11—C12—C13—N1	179.06 (15)
Br1—C3—C4—C5	178.77 (11)	C11—C12—C13—C8	−0.2 (2)
C3—C4—C5—C6	0.6 (2)	N2—C8—C13—N1	0.23 (16)
C4—C5—C6—C1	−0.4 (2)	C9—C8—C13—N1	−179.16 (14)
C4—C5—C6—C7	−177.66 (14)	N2—C8—C13—C12	179.59 (13)
C2—C1—C6—C5	0.3 (2)	C9—C8—C13—C12	0.2 (2)
C2—C1—C6—C7	177.85 (13)	C15—O1—C14—O2	1.0 (2)
C13—N1—C7—N2	−0.29 (17)	C15—O1—C14—C11	−179.41 (13)
C13—N1—C7—C6	177.75 (13)	C12—C11—C14—O2	1.2 (2)
C8—N2—C7—N1	0.43 (17)	C10—C11—C14—O2	−179.21 (15)
C17—N2—C7—N1	176.78 (14)	C12—C11—C14—O1	−178.39 (13)
C8—N2—C7—C6	−177.48 (14)	C10—C11—C14—O1	1.2 (2)
C17—N2—C7—C6	−1.1 (2)	C14—O1—C15—C16	173.90 (14)
C5—C6—C7—N1	150.20 (15)	C7—N2—C17—C18	100.34 (17)
C1—C6—C7—N1	−27.1 (2)	C8—N2—C17—C18	−83.90 (17)
C5—C6—C7—N2	−32.0 (2)	N2—C17—C18—C19	−170.67 (12)
C1—C6—C7—N2	150.63 (15)	C20—N3—C19—C18	75.0 (2)
C7—N2—C8—C9	178.93 (16)	C22—N3—C19—C18	−105.67 (18)
C17—N2—C8—C9	2.3 (2)	C17—C18—C19—N3	68.15 (17)
C7—N2—C8—C13	−0.38 (15)	C21—N4—C20—N3	0.10 (19)
C17—N2—C8—C13	−176.99 (13)	C22—N3—C20—N4	−0.28 (19)
N2—C8—C9—C10	−179.58 (15)	C19—N3—C20—N4	179.12 (14)
C13—C8—C9—C10	−0.4 (2)	C20—N4—C21—C22	0.1 (2)
C8—C9—C10—C11	0.5 (2)	N4—C21—C22—N3	−0.3 (2)
C9—C10—C11—C12	−0.5 (2)	C20—N3—C22—C21	0.35 (19)
C9—C10—C11—C14	179.95 (14)	C19—N3—C22—C21	−179.05 (15)
C10—C11—C12—C13	0.3 (2)		

### Hydrogen-bond geometry (Å, °)

**Table:**

*D*—H···*A*	*D*—H	H···*A*	*D*···*A*	*D*—H···*A*
O1W—H2W1···N4^i^	0.92 (3)	1.99 (3)	2.910 (2)	175 (3)
O1W—H1W1···N1^ii^	0.81 (3)	2.16 (3)	2.891 (2)	151 (2)
C17—H17B···O1W^iii^	0.99	2.41	3.236 (2)	141.
C19—H19B···O1W^iii^	0.99	2.56	3.327 (2)	135.
C20—H20A···O2^iv^	0.95	2.58	3.301 (2)	133.

Symmetry codes: (i) −*x*+1, −*y*, −*z*+1; (ii) *x*+1/2, −*y*+1/2, *z*−1/2; (iii) *x*+1/2, −*y*+1/2, *z*+1/2; (iv) −*x*+1/2, *y*−1/2, −*z*+3/2.
